# The preterm social brain: altered functional networks for Theory of Mind in very preterm children

**DOI:** 10.1093/braincomms/fcaa237

**Published:** 2021-01-25

**Authors:** Sarah I Mossad, Marlee M Vandewouw, Mary Lou Smith, Margot J Taylor

**Affiliations:** 1 Department of Psychology, Hospital for Sick Children, Toronto, ON M5G 1X8, Canada; 2 Department of Diagnostic Imaging, The Hospital for Sick Children, Toronto, ON M5G 1X8, Canada; 3 Neurosciences & Mental Health, SickKids Research Institute, Toronto, ON M5G 0A4, Canada; 4 Department of Psychology, University of Toronto, Toronto, ON M5S 3G3, Canada

**Keywords:** preterm birth, magnetoencephalography (MEG), fMRI, Theory of Mind, functional connectivity

## Abstract

Neurodevelopmental difficulties emerge in very preterm born children (<32-week gestation) in infancy and continue to early adulthood but little is known about their social-cognitive development. This study utilized the complementary methodological advantages of both functional MRI and magnetoencephalography to examine the neural underpinnings of Theory of Mind in very preterm birth. Theory of Mind, one of the core social-cognitive skills, is the ability to attribute mental states to others, and is crucial for predicting others’ behaviours in social interactions. Eighty-three children (40 very preterm born, 24 boys, age = 8.7 ± 0.5 years, and 43 full-term born, 22 boys, age = 8.6 ± 0.5 years) completed the study. In functional MRI, both groups recruited classic Theory of Mind areas, without significant group differences. However, reduced Theory of Mind connectivity in the very preterm born group was found in magnetoencephalography in distinct theta, alpha and beta-band networks anchored in a set of brain regions that comprise the social brain. These networks included regions such as the angular gyrus, the medial pre-frontal cortex, the superior temporal gyrus and the temporal poles. Very preterm born children showed increased connectivity compared to controls in a network anchored in the occipital gyri rather than classical social-processing regions. Very preterm born children made significantly more attribution errors and mis-construed the social scenarios. Findings offer novel insight into the neural networks, supporting social cognition in very preterm born children and highlight the importance of multimodal neuroimaging to interrogate the social brain in clinical populations.

## Graphical Abstract

**Graphical Abstract:**
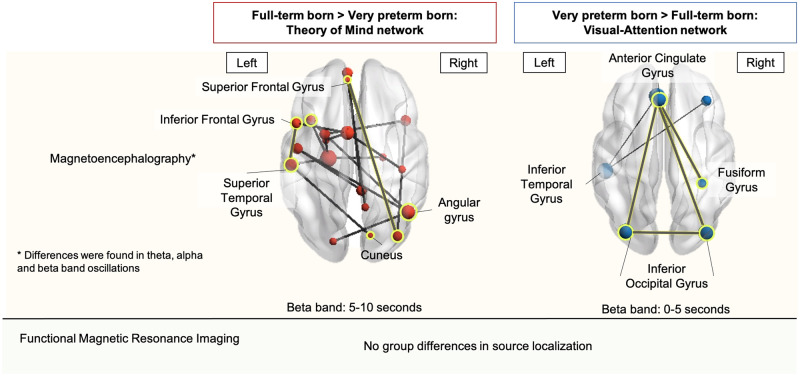

## Introduction

Very preterm birth [VPT, ≤32 weeks of gestational age, (GA)] occurs during one of the most critical periods of brain development. During this time, early sensory processing in the brain is dependent on the expansion of thalamocortical afferents in the cortical plate followed by the development of cortico-cortical fibres in the neonatal period (Kostović and Jovanov-Milosˇević, 2006). Approximately, 15 million babies are born preterm around the world each year and of those 10% are born VPT. Advancements in neonatal medicine have reduced the lower limit of GA at which a foetus is viable from 28-week GA in the 1980s to 22- to 23-week GA currently (Hopp and Baron, 2017; Chawanpaiboon *et al.*, 2019; Silveira *et al.*, 2019). This steady increase in survival rates has been accompanied by an increase in neurodevelopmental morbidities including lifelong difficulties in social functioning such as more peer problems, social withdrawal and fewer successful close relationships (Hille *et al.*, 2008; Moster *et al.*, 2008; Jones *et al.*, 2013; Ritchie *et al.*, 2015) but little is known about whether altered neural processing of social information underlies the expression of these social skill deficits.

We addressed this question by examining both the functional localization (using functional magnetic resonance imaging, fMRI) and the functional connectivity (using magnetoencephalography, MEG) of the social brain along with neuropsychological and behavioural assessments in 8-year-old children born VPT who were age and sex matched with full-term born (FT) children (≥38-week GA) (total, *n* = 83). The social brain is a term used to describe the set of brain regions that are consistently recruited for processing information about others’ intentions, thoughts, emotions and behaviour. These regions include the medial pre-frontal cortex, temporal–parietal junction, superior temporal gyrus and amygdalae (Saxe and Wexler, 2005; Carrington and Bailey, 2009; Gweon *et al.*, 2012). Since VPT birth is a risk factor for neurodevelopmental disorders (Bröring *et al*., 2018), establishing whether VPT birth is associated with atypical connectivity in the social brain could help generate subsequent research, comparing preterm-born children with or without a neurodevelopmental disorder.

We interrogated the social brain using a Theory of Mind (ToM) task. ToM is the ability to make mental representations of the mental states of others (including their beliefs, thoughts and intentions) and understand that these mental states are independent from one’s own (Premack and Woodruff, 1978). Explicit ToM abilities substantially improve in the pre-school years (4–5 years) and continue to mature throughout adulthood (Wellman *et al.*, 2001). There is a positive, bidirectional relationship between mastery of ToM skills and peer acceptance, prosocial behaviour and developing friendships (Lagattuta *et al.*, 2014), making ToM a particularly important skill to assess in VPT children. The ToM task used in this study measures social attribution. The task is based on a procedure first introduced by Heider and Simmel (1944), and involves videos of moving shapes, depicting complex social interactions that can be encountered in daily life. This task has been shown to recruit regions of the social brain and was preferentially utilized due to its low cognitive and language demands, making it ideal for use with paediatric populations (Castelli, 2002; Schultz *et al.*, 2003; Gobbini *et al.*, 2007).

Taking advantage of the superior spatial resolution of fMRI and the remarkable temporal resolution of MEG, we captured the hemodynamic activations related to ToM and real-time functional connectivity (indexed by the amplitude envelope correlation) in the ToM network, respectively. Network dynamics provide information about moment-by-moment interactions among ToM regions and therefore will most accurately characterize how children respond to the demands of their external environment. We predicted atypical neural processing during this social attribution task in the VPT group compared to FT children, marked both by atypical source localization (measured in fMRI) and atypical functional network connectivity (measured in MEG).

## Materials and methods

### Participants

In total, 105 babies born VPT were recruited from the Neonatal Intensive Care Unit at the Hospital for Sick Children, Toronto, Canada. Neuroimaging was completed within 2 weeks of birth, with follow-up imaging at term then again at 2, 4, 6 and 8 years. At 8 years, all participants from the 4-year cohort were contacted and 40 VPT children returned to complete the assessments. The returning subset of participants did not differ from the original cohort (*n* = 105) on GA or on any neonatal assessment scores (Supplementary Table 1). Therefore, this sample of VPT children was not particularly ‘healthier’ based on these neonatal medical assessments compared to those who did not return. A FT control group (GA, ≥37 weeks) was recruited to match the VPT born group on age and sex by posted advertisements around the hospital and through flyers posted at schools and social media. At 8 years, 43 FT participants took part in the study.

Groups did not differ on age, sex, handedness, mean neighbourhood income or mother’s education but VPT children had lower birth weight (*P *<* *0.001), GA (*P *<* *0.001) and IQ (*P* < 0.001, Table 1). Since this difference in IQ may be a confounding variable, we also tested if IQ predicted any group differences in neuroimaging. There was a potential for selection bias where families of FT children with higher IQ self-selected to participate in this intensive 4-h long study. Successful MRI and MEG scanning were completed in 78 children (38 in VPT and 40 in FT).

**Table 1:** Group characteristics

	VPT (*n* = 40)	FT (*n* = 43)	*t/χ* ^2^	*P*-value
Age (years)	8.7 ± 0.51	8.6 ± 0.46	*t*(78) = 1	0.27
Sex (F:M)	16F:24 M	21F:22 M	*χ* ^2^(1) = 0.5	0.47
Birth weight (kg)	1.2 ± 0.22	3.5 ± 0.60	*t*(54) = 24	<0.001
Gestational age (weeks)	29 ± 1.41	39 ± 1.35	*t*(67) = 27	<0.001
IQ	105.65 ± 14.5	119.7 ± 11	*t*(72) = 5	<0.001
Mean neighbourhood income ($)	111 522	121 096	*t*(75) = 1	0.32
Mother’s education	*n* = 39 Grade school (*n* = 1) High school (*n* = 5) Some post-secondary training (*n* = 7)^a^ University/College (*n* = 18) Post-graduate training (*n* = 8)	*n* = 41 High school (*n* = 1) Some post-secondary training (*n* = 7)^a^ University/College (*n* = 18) Post-graduate training (*n* = 15)	χ^2^(5)=6.7	0.24

a Some post-secondary training: includes trade programs and diplomas that do not include university or college.

Exclusion criteria included intellectual impairment or any other language or vision issues, preventing successful completion of tasks as well as standard MEG/MRI contraindications. This study was approved by the SickKids research ethics board. Informed assent was obtained from participants and written consent from the parents according to the declaration of Helsinki.

### Assessments

Children completed the vocabulary and matrix-reasoning subtests of the Wechsler Abbreviated Scale of Intelligence (Wechsler, 1999) as an estimate of their Full-Scale IQ. Parents completed the social responsiveness scale (SRS), which is most frequently used in screening of autistic features in autism spectrum disorder populations and was used here to assess social functioning (Constantino, 2013). Since cognitive and social-cognitive processes are tightly linked, parents also completed the Behavior Rating Inventory of Executive Function (Gioia *et al.*, 2000) to test whether executive functioning difficulties could be ruled out as a driving factor for the observed social difficulties.

### The social attribution task

Heider and Simmel (1944) first showed that kinetic features of moving shapes affected how they were perceived, such that participants attributed mental states, intentions, perspectives and beliefs to the shapes. The social attribution task was designed by Klin (2000) and Schultz *et al.* (2003) and includes conditions that stimulate participants to make social attributions to the moving shapes.

**Figure 1:**
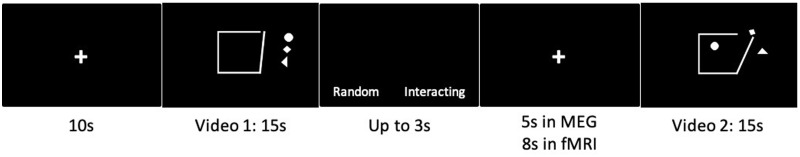
**Social attribution task protocol.** A 10-s baseline was presented at the beginning of each run. Each 15-s video was then presented. Participants were then given 3 s to respond to whether the shapes were moving randomly (physical condition) or interacting (social condition) followed by an inter-stimulus interval. The protocol was the same in MEG and fMRI, except in the fMRI the inter-stimulus interval was 8 s, whereas it was 5 s in the MEG. Task was designed by Klin (2000) and Schultz *et al.* (2003).

The social attribution task involved 15-s videos. Three white shapes, a circle (diameter, 1 cm), a square/diamond (0.6 × 0.6 cm) and a triangle (1.4 × 1 × 1 cm), moved against a black background, whereas a fixed white frame (5 × 5 cm) was centred in the middle of the screen (Fig. 1) with one side hinged at the bottom right corner occasionally opening and/or closing. This task consisted of two conditions: a social condition and a physical (control) condition. In the social condition, the videos portrayed a story evolving from the start to the end of the video. Participants were instructed to attend to the entire 15 s. In the control condition, each shape’s movement was random (not contingent upon other shapes’ movement), making the videos unlikely to be perceived as social interactions; the shapes were therefore described based on their physical properties. While the type of movement was by definition different between social and physical conditions, the basic visual characteristics (speed, orientation of motion, etc.) were similar.

Children first practiced the task on two videos not used in the scanners. In both the MEG and the fMRI scans, a 10-s baseline period was presented at the beginning followed by the video (social or physical). Immediately following each video, a fixation cross was presented in the centre of the screen with two words presented on opposite sides of the screen (4 cm below fixation cross and 11 cm apart). Participants had up to 3 s to respond by button press whether they thought the shapes were ‘Interacting’ or ‘Random’. After a response or if 3 s had passed, the fixation cross remained on the screen without the words for an additional 5 s in the MEG and 8 s in the fMRI. Following this ISI, the next video was then presented. The eight videos were presented in pseudo-random order for a total run time of about 3.3 min in the MEG and 3.7 min in the fMRI for each run. The task was run three times in each modality for a total of 12 social video presentations and 12 physical video presentations in each neuroimaging modality.

### Social attribution task description

A description of the videos was obtained from each child after the MEG and fMRI scanning were done. Two VPT children and one FT child refused to complete this final component of the study due to fatigue. Descriptions were obtained in 36 VPT and 42 FT children. Each participant watched the 15-s videos as many times as they needed to respond to the prompt ‘Tell me everything the shapes are doing here’. The children’s descriptions were obtained using a voice recorder and then scored for the type of social attributions and the type of errors made, based roughly on instructions by Klin (2000).

### Functional magnetic resonance imaging acquisition and pre-processing

MRI data were collected on a 3.0T Siemens Prisma scanner with a 20-channel head and neck coil. T1-weighed structural MRI images were collected using a 3D MPRAGE sequence (TR/TE/TI: 1870/3.14/945 ms; FA: 9°; field of view: 240 × 256 mm; number of slices: 192; resolution: 0.8 mm isotropic; scan time: 5:01 min). Functional images were acquired during the social attribution task with an echo planar imaging sequence (TR/TE: 1500/30 ms; FA: 70°; field of view: 222 x 222 mm; number of slices: 50; resolution: 3 mm isotropic). The mean number of volumes/run was 140 ± 3.9. The task was commenced automatically after scanner stabilization. All scanning took place at the Hospital for Sick Children (Toronto, Canada).

fMRI data were pre-processed in a locally developed pipeline using standard AFNI (https://afni.nimh.nih.gov), FreeSurfer (Dale *et al.*, 1999) and FSL (https://www.fmrib.ox.ac.uk/fsl) tools. Each subject’s T1-weighted image was skull stripped using FreeSurfer. Task data were slice time and motion corrected and estimates of the six motion parameters (3 translations + 3 rotations) were obtained. Frame-wise displacement was calculated, and volumes with frame-wise displacement of >0.9 mm were removed from the data (Siegel *et al.*, 2014). Runs where more than 1/3 of the volumes were scrubbed were excluded, and participants with only two remaining runs were excluded from the analysis. After exclusion, the final number of participants in each group was 25 VPT children and 35 FT children. The reasons for exclusion from the original cohort were excessive motion, noise in the data or due to participants refusing to complete the fMRI portion of the study due to stress or anxiety (children were allowed to watch a movie of their preference during acquisition of other MRI sequences). In the fMRI, the mean framewise displacement in the data analysed was 0.65 mm in the VPT group and 0.45 mm in the FT; this difference was not significant between groups. The average number of low motion frames retained in each group was: 131.4 ± 7.7 in the VPT group and 127.4 ± 12.7 in the FT group. The number of low motion frames retained per subject is available upon request.

Data were smoothed with a 6-mm FWHM Gaussian kernel, intensity normalized and signal contributions from the white matter, CSF and whole-brain, along with the six motion parameters were regressed from the data. Data were then bandpass filtered from 0.01 to 0.2 Hz. Functional images were linearly registered to MNI standard space with FSL’s FLIRT (Jenkinson and Smith, 2001). Independent component analysis denoizing was performed via FSL’s FIX (Griffanti *et al.*, 2014; Salimi-Khorshidi *et al.*, 2014), trained on 20 subjects distributed evenly over the VPT and FT groups by two authors M.M.V. and S.I.M.

### Magnetoencephalography acquisition and pre-processing

Participants’ MEG scans were obtained on a 151-channel CTF system in a magnetically shielded room in supine position (CTF MEG International Service LP, Coquitlam, BC, Canada). MEG recordings were obtained at a 600-Hz sampling rate and third-order noise cancellation was applied. Head localization was performed continuously throughout the recording. Stimuli were presented on a rear-projection screen ∼80 cm from the participant. Responses were collected on a VPIXX 4 button pad (Visual Science Solutions, Saint-Bruno, Canada).

All MEG data processing, analyses and statistics were conducted in MATLAB using custom scripts implementing functions from the FieldTrip toolbox (Oostenveld *et al.*, 2011), Network-Based Statistics (Zalesky *et al.*, 2010), BrainNetViewer (Xia *et al.*, 2013) and Marc’s MEG Mart (MMM; https://gitlab.com/moo.marc/MMM).

MEG data were epoched at the onset of the ‘Social’ and ‘Physical’ trials from −5 to 17 s. Independent component analysis was used to choose and reject components with heartbeat and ocular artefacts. Components were rejected based on visual analysis. Trials were also rejected if the signal exceeded 2500 fT. Visual inspection of each trial for each participant was done by author SIM. Head motion rejection was achieved by fitting a rigid sphere to the average fiducial marker locations (nasion, left and right pre-auricular points) and tracking the translation and rotation of the sphere continuously throughout the recording using the HeadMotionTool from the MMM toolbox. The median head position was used as a reference and trials with >10 mm motion were rejected. The number of participants remaining, following exclusion due to headmotion, was 30 VPT and 31 FT children. The average number of good quality trials in each group was 22 trials in the VPT group and 22.2 trials in the FT group.

### Source estimation

MEG data were imported into MATLAB using FieldTrip and were mean-centred and filtered with a 4th order Butterworth band-pass filter at 1–150 Hz and a discreet Fourier transform notch filter at 60 and 120 Hz to remove line noise. Subject-specific single-shell head models based on the MRI from each subject were computed using SPM12 through FieldTrip and template coordinates were non-linearly transformed into subject-specific coordinates. Linearly constrained minimum variance beamforming with 5% regularization and projection of the activity to the dominant orientation was performed to estimate the neural activity index at each source coordinate.

### Connectivity analysis

Linearly constrained minimum variance source estimation was performed at the centroid of each of the cortical and sub-cortical regions of the Automated Anatomical Labeling atlas, and the timeseries were filtered into four canonical frequency bands: theta (4–7 Hz), alpha (8–12 Hz), beta (13–29 Hz), gamma (30–55 Hz) and using FIR filters (MATLAB’s fir1).

A symmetric orthogonalization procedure (Colclough *et al.*, 2015) was applied to the filtered neural activity index time series to remove the effects of signal leakage. Amplitude envelopes were computed using the absolute value of the Hilbert transform (Brookes *et al.*, 2011; Hipp *et al.*, 2012). To obtain the amplitude envelope correlations (AEC), the Pearson’s correlation coefficient was computed for amplitude envelopes from each pair of nodes. The AEC time series were baselined by computing the fractional change from the mean baseline AEC from −5 to 0 s. AEC was chosen over other measures of synchrony as it has the lowest susceptibility to co-registration-related errors (Liuzzi *et al.*, 2017).

### Statistical analysis

#### Functional magnetic resonance imaging

In a time-series (first-level) analysis using FMRIB’s Improved Linear Model (Woolrich *et al.*, 2001), the task conditions (social, physical, baseline and response) were used as explanatory variables and convolved with a double-gamma hemodynamic response function. The model included nuisance regressors for the six motion parameters and scrubbed volumes and investigated contrasts between the social and the physical conditions. Second-level analysis was performed to average contrast estimates over runs within each subject using FSL’s FEAT with fixed effects (Woolrich *et al.*, 2001). Group-level analysis was performed using FMRIB’s Local Analysis of Mixed Effects (Woolrich *et al.*, 2004). Two-sample unpaired *t*-tests were used to assess differences between VPT and FT, with mean frame-wise displacement as a covariate. Gaussian Random Field theory was used for cluster-level multiple comparisons correction, with *Z* > 3.1 and a corrected cluster significance threshold *P *=* *0.05.

#### Magnetoencephalography

Whole-brain network connectivity differences between groups in the social condition VPT > FT and FT > VPT (*P < 0.05*) were identified in Network-Based Statistics (Zalesky *et al.*, 2010). Network-Based Statistics applies mass univariate testing to test the null hypothesis at each connection. In this study, a strict *t*-threshold of 2.5–3.0 was applied, allowing only connections with a *t*-value at or above 2.5 to be included in subsequent analyses. Network-Based Statistics then examines the topology among connections passing supra-threshold connections using cluster-based statistical methods. Each cluster is composed of supra-threshold connections, with a path connecting any two nodes. Finally, a family-wise error rate-corrected *P*-value is computed for each component using permutation testing (a non-parametric computation). Permutations were repeated 5000 times. *P*-values for the resulting networks in each contrast (VPT versus FT) are reported across the three time windows (0–5, 5–10 and 10–15 s).

#### Behavioural data

Behavioural data and psychological assessments were analysed in R (R Development Core Team & R Core Team, 2018). Between-group differences in sex and mother’s education were analysed with *χ*^2^ tests. Between-group differences in age, average neighbourhood income, GA and birthweight were analysed using two-tailed independent samples *t*-tests. *P*-values were reported significant at *P *<* *0.05. Descriptions of the videos were scored on three separate measures: category, error type and word count. Two-tailed independent samples *t*-tests were used to assess group difference, controlling for FDR (Benjamini and Hochberg, 1995) for condition type (social versus physical). Group differences on the sub-scales of the SRS are the Behavior Rating Inventory of Executive Function and were also analysed using two-tailed independent samples *t*-test, controlling for multiple comparisons (4 for SRS and 3 for Behavior Rating Inventory of Executive Function, 7 in total) using FDR correction. Participants with missing data were excluded from the analysis and sample sizes are indicated for each analysis.

#### Brain–behaviour analysis

To explore the association between MEG connectivity and errors, we investigated whether Group × Response Errors predicted mean node strength using generalized linear models in R. Node strength was computed by taking the sum of the AEC connectivity values at each node of the observed networks. For each subject, mean strength of the observed network was derived by taking the average of node strength values. We controlled for multiple comparisons across networks and frequency bands using FDR correction.

### Data availability

The data that support the findings of this study are available from the corresponding author, upon reasonable request.

## Results

### Behavioural results

**Figure 2:**
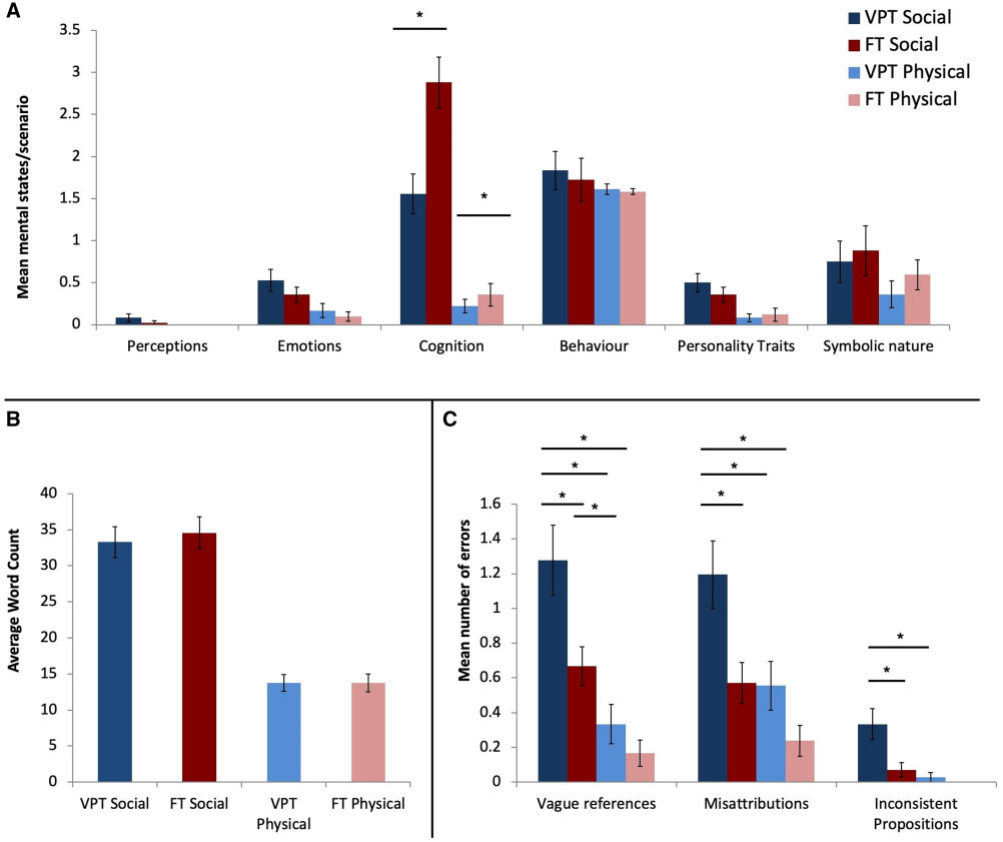
(**A**) The mean number of attributions in VPT and FT born children across scenarios, showing social interactions compared to random physical motion. FT children attributed more cognitive states (thoughts, wants and needs) compared to VPT children on the videos showing social interaction. Error bars represent standard error (SE). (**B**) Average word count in social and physical motion in each of the groups (±SE). (**C**) More errors were made by VPT than FT children in descriptions of scenarios involving social interactions and random physical movement (±SE).

There were no significant differences between groups in accuracy or reaction time in either condition in the MEG or fMRI. After neuroimaging, each child re-watched the videos and described each one. The narratives were scored based on some of the criteria described in Klin (2000). Attributions were categorized into perceptions (e.g. circle *notices* square), emotions (e.g. square is *mad*), cognition (e.g. triangle *wants* to open the door), behaviour (e.g. triangle *tricks* square), personality traits/personal relations (e.g. triangle is *mean*) and symbolic nature (e.g. they put square in *prison*). Group differences were found only in attributions pertaining to cognitive states of the shapes (*P*_*FDR*_ = 0.0045), where FT children attributed significantly more mental state words such as wants, needs and thoughts compared to VPT children (Fig. 2A). There were no differences in word count between groups for either social or physical stimuli (Fig. 2B).

The type of errors was also scored and categorized into: vague references, misattributions and inconsistent propositions, an example of each error is listed in Supplementary Table 2. Vague references refer to descriptions where important information such as the subject, object or their relationship is missing. Misattributions indicate errors made due to making incorrect attributions (e.g. the triangle is *helping* the square is a misattribution in a scenario where the triangle is *deceiving* the square). Finally, any inconsistencies that render the description incoherent were also recorded. VPT children gave more vague descriptions [*t*(55) = 2.6, *P*_*FDR*_ = 0.02], made more misattribution errors [*t*(58.9) = 2.7, *P*_*FDR*_ = 0.02], and had more inconsistent details [*t*(48)=2.6, *P*_*FDR*_ = 0.02] (Fig. 2C). Children in both groups made more errors, describing the social interactions compared to random physical motion.

### Neuroimaging results: fMRI results

**Figure 3:**
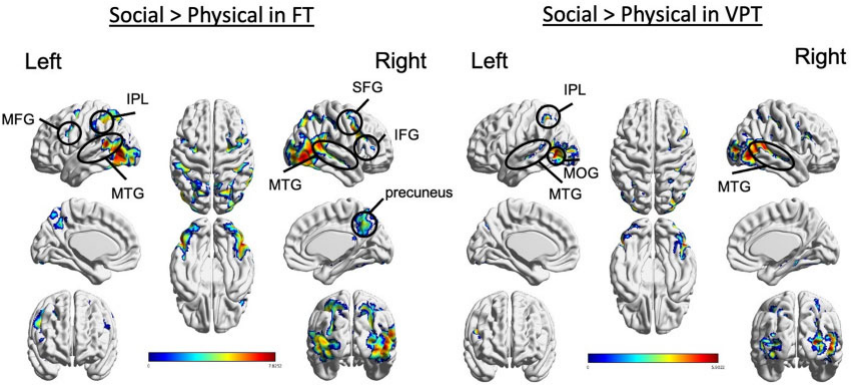
**fMRI activations in the Social>Physical contrast within each group.** There were no significant group differences. MFG, middle frontal gyrus; MTG, middle temporal gyrus; IPL, inferior parietal lobule; SFG, superior frontal gyrus; IFG, inferior frontal gyrus; MOG, middle occipital gyrus.

Scenarios depicting social interactions compared to scenarios depicting random physical motion (Social>Physical) elicited fMRI activity in regions that comprise the ToM network within each group, as reported in adults (Gobbini *et al.*, 2007). There were no significant group differences in BOLD-related activations (Fig. 3). Regions activated for the Social>Physical contrast included the superior frontal gyri, inferior frontal and left superior and inferior parietal lobules (Supplementary Table 3).

### MEG results

**Figure 4:**
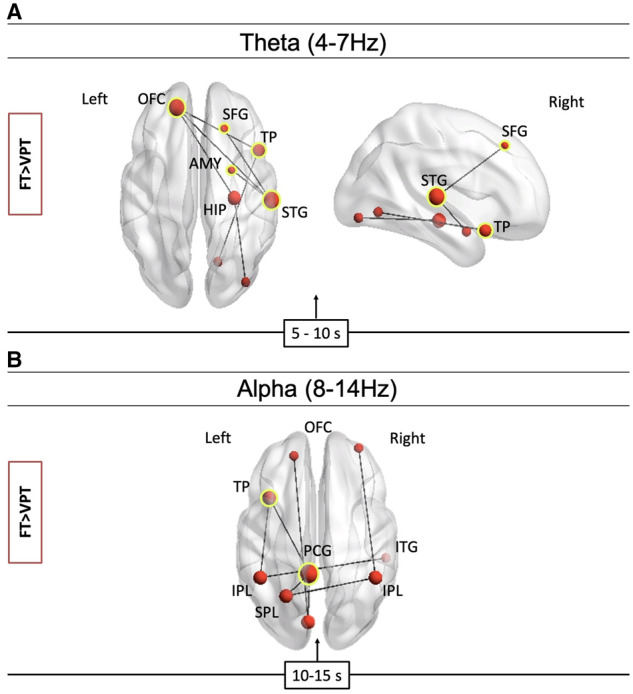
**Reduced connectivity in the VPT group compared to the FT group in theta and alpha bands.** A right-lateralized frontal–temporal network was found in theta band (**A**), whereas a bilateral parietal–occipital network was found in alpha band (**B**). OFC, orbital frontal cortex; SFG, superior frontal gyrus; AMY, amygdala; HIP, hippocampus; PCG, posterior cingulate gyrus; TP, temporal pole; STG, superior temporal gyrus; ITG, inferior temporal gyrus; IPL, inferior parietal lobule; SPL, superior parietal lobule.

In contrast to the fMRI, we observed remarkable differences in functional connectivity between groups across time and frequency in MEG in the social condition. From 5 to 10 s, reduced connectivity in the VPT group was found in a right-lateralized network in *theta band* (Fig. 4A) anchored in the right superior temporal gyrus, right temporal pole and the left orbital frontal gyrus, *P *=* *0.03. From 10 to 15 s, reduced connectivity was found in the VPT group in a network in *alpha band* anchored in the posterior cingulate gyrus, the left superior parietal gyrus, bilateral parietal lobules and left temporal pole (*P *=* *0.01). (Fig. 4B).

**Figure 5:**
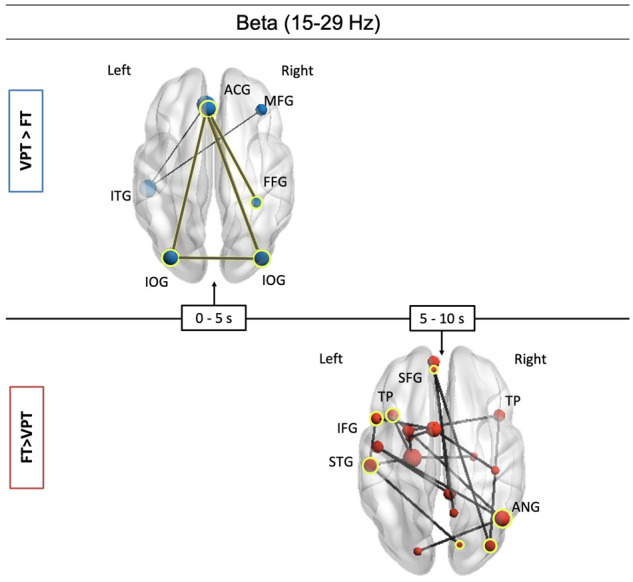
**Between-group differences in beta band.** Top panel: Increased connectivity in the VPT group in beta band (0–5 s), in a small network in the bilateral occipital gyrus and anterior cingulate gyrus. Bottom panel: Reduced connectivity in the VPT group was found (5–10 s), in a network involving classical ToM regions, such as the right-angular gyrus, medial pre-frontal cortex, orbital frontal gyrus and left superior temporal gyrus. ACG, anterior cingulate gyrus; MFG, middle frontal gyrus; SFG, superior frontal gyrus; IFG, inferior frontal gyrus; TP, temporal pole; FFG, fusiform gyrus; STG, superior temporal gyrus; ANG, angular gyrus; IOG, inferior occipital gyrus.

The most notable findings were the extent of difference in networks employed during the social condition in the *beta band*. From 0 to 5 s, VPT children activated a sparse network, compared to FT children, that included the right and left occipital gyri, the right fusiform gyrus and the left anterior cingulate gyrus (Fig. 5, top panel, *P *=* *0.008). Increased connectivity in the FT group was found in beta from 5 to 10 s (Fig. 5, bottom panel, *P *=* *0.03). This network included classic ToM areas; it was anchored in the right angular gyrus, left superior temporal gyrus and parahippocampal gyrus, and included the left superior and inferior frontal gyri and bilateral temporal poles.

### Brain–behaviour associations

We found that an interaction between Group × Error Type was associated with network connectivity in alpha band. Specifically, misattribution errors were associated with reduced mean network connectivity in the alpha band network shown in Fig. 4B in the VPT group, *P*_FDR_ = 0.002. Additionally, inconstant propositions were associated with reduced connectivity in the beta band social-cognitive network (Fig. 5, bottom panel, *P*_FDR_ = 0.007) in the VPT group. We did not find any association between error type and network connectivity in the theta band network.

### Assessments

The VPT group had an average IQ of 105.7 (average range) and the FT group had an average IQ of 119.7 (high average range). There were no group differences on parent ratings of executive functioning or social responsiveness. However, more VPT children met criteria for clinically elevated difficulties on social cognition but not executive functioning (Table 2). A cut-off *T*-score of 60 for both the SRS and the Behavior Rating Inventory of Executive Function was used to indicate elevated scores in each measure.

**Table 2:** Neuropsychological outcome in VPT and FT children

	VPT (*n* = 40)	FT (*n* = 43)	*t, P*	VPT (*n* = 40)	FT (*n* = 43)	*χ*_2,_ ***P***
Full-scale IQ	105.7 ± 14	119.7 ± 11	*<0.01*			
Social-responsiveness scale				Clinically significant cases		
Social awareness	51.9 ± 9.8	48.1 ± 9.15	*t*(79) = 1.8, *P* = 0.06	*n* = 8	*n* = 4	*χ*_2_(1) = 1.24, *P*_FDR_ = 0.2
Social cognition	48.9 ± 8.9	46.6 ± 5.4	*t*(62) = 1.3, *P* = 0.17	*n* = 7	*n* = 0	*χ*_2_(1) = 6.2, *P*_FDR_ = 0.04
Social communication	48.4 ± 8.3	46.7 ± 6.5	*t*(73) = 1, *P *= 0.3	*n* = 6	*n* = 2	*χ*_2_(1) = 1.58, *P*_FDR_ = 0.2
Social motivation	50.0 ± 9.3	46.7 ± 6.7	*t*(70) = 1.8, *P *= 0.06	*n* = 6	*n* = 2	*χ*_2_(1) = 1.58, *P*_FDR_ = 0.2
Behaviour rating inventory of executive function				Clinically significant cases		
Behaviour regulation index	48.2 ± 10	46.5 ± 7.8	*t*(72) = 0.65, *P *= 0.5	*n* = 3	*n* = 0	*χ*_2_(1) = 1.59, *P* = 0.2
Metacognitive index	48.6 ± 9.0	46.3 ± 8.4	*t*(81) = 0.9, *P *= 0.33	*n* = 3	*n* = 0	*χ*_2_(1) = 1.59, *P* = 0.2

## Discussion

There is increasing evidence that VPT children experience difficulties in acquiring and appropriately utilizing their social skills (Montagna and Nosarti, 2016; Fenoglio *et al.*, 2017) and our results demonstrate important differences in ToM processing, which may underlie their challenges in everyday social functioning. This study is the first to examine the neural underpinnings of ToM, utilizing methodological advantages from both fMRI and MEG in VPT children.

Our results suggest that FT children are able to meet the environmental demands of their social milieu by employing neural networks that include regions of the social brain modulated by theta, alpha and beta neural oscillations. Although VPT children relied on a network in beta band as well, this network emerged earlier and was anchored in visual attention regions. These connectivity differences offer an explanation for difficulties that VPT children may face during complex social interactions that require honed brain network dynamics. It was also significant that no group differences were recorded at the source localization level measured in fMRI. Our fMRI results demonstrate that the preterm social brain developed the expected functional specialization for this ToM task, but MEG showed that these ToM regions are characterized by strikingly different functional connectivity networks.

False-belief tasks are generally regarded as the gold standard for ToM in typical development (Ruffman, 2014). False belief describes a scenario where one individual understands that another has a belief incongruent with reality. In this case, the individual holds two opposing mental representations: his/her own and another’s. Our group has previously shown that 7- to 12-year-old VPT children with average IQ exhibit significantly worse performance on a false-belief task compared to controls (Mossad *et al.*, 2017). Although false-belief tasks have been widely used, some factors make it less practical for use particularly with young clinical populations who have difficulties in cognitive functioning. Clinical populations with verbal comprehension difficulties may also perform worse due solely to complex task instructions compared to others with average or superior language skills (Klin, 2000). The social attribution videos were designed so that each scenario evokes the sense of a real-life event that allows participants to employ social-cognitive processes without requiring pre-requisite cognitive skills.

Descriptions of the scenarios in the social attribution task also provided information regarding the nature of social-cognitive problems rather than dichotomizing social-cognitive pathologies into ToM or non-ToM categories. VPT children did not differ from FT children on measures of accuracy or reaction time when they were asked to categorize each scenario as either social or non-social. However, FT children made more cognitive state attributions compared to VPT children, the only domain where the groups differed. Wellman *et al.*, (2011) suggested that there is a gradual order for acquisition of various ToM skills, with more difficult mental-state attributions developing last. Therefore, it is possible that VPT children’s cognitive state attribution development is delayed compared to their FT peers, a hypothesis proposed in a previous study (Williamson and Jakobson, 2014). Based on these differences, we conclude that at more coarse and categorical querying, VPT children are able to distinguish whether a scenario depicted a social interaction. However, they misinterpreted that the details (misattribution errors) did not give clear accounts of who the agents were and how they behaved (vague references), and contradicted facts reported in their descriptions (inconsistent propositions). Consequently, VPT children may be prone to making these errors while interpreting social interactions, which can result in difficulties navigating their social environment. Additionally, we found that 17.5% of our sample of VPT children met criteria for clinically elevated social-cognitive difficulties, despite average executive functioning skills. Similar prevalence rates have been reported by Korzeniewski *et al.* (2017) who found that 16% of their sample of average IQ, 10-year-old preterm children (*n* = 628) met clinical criteria for problems on the SRS. They also found that worse social responsiveness symptomology on the SRS was not related to worse inhibition scores, further supporting our findings that there can be specific difficulties in the social domain in preterm populations without significant executive functioning problems. One clinical implication is that consistent screening, assessment and follow-up of social-cognitive skills should be implemented in VPT children into school age.

fMRI studies have revealed a neural network associated with social attribution (Schultz *et al.*, 2003; Gobbini *et al.*, 2007; Castelli *et al.*, 2013). This network includes the medial pre-frontal cortex, associated with evaluating one’s own thoughts (Amodio and Frith, 2006), the amygdalae and the superior temporal gyri/sulci, recruited in biological and intentional motion (Allison *et al.*, 2000; Martin and Weisberg, 2003; Schultz *et al.*, 2003, 2004, 2005; Blake and Shiffrar, 2007; Gobbini *et al.*, 2007; Tavares *et al.*, 2008; Santos *et al.*, 2010). Other regions include the insula and the posterior cingulate cortex, the right inferior temporal and fusiform gyri, the inferior frontal gyri. We did not find group differences in the regions recruited in the social condition measured by fMRI. This important finding establishes that the ToM network is present in VPT children.

We found that ToM is supported by several networks of neural oscillations in theta, alpha and beta frequencies; beta band is associated with top-down processing and alpha is often associated with attention and memory processing, whereas theta reflects long-range communication in the brain (Von Stein and Sarnthein, 2000; Engel and Fries, 2010; Palva *et al.*, 2010; Klimesch, 2012; Mellem *et al.*, 2013; Sherman *et al.*, 2016; Solomon *et al.*, 2017; Betti *et al.*, 2018; Richter *et al.*, 2018; Soto-Icaza *et al.*, 2019). Thus, the involvement of all three frequency bands is consistent with earlier work, showing the complexity of the neural systems underlying this sophisticated cognitive skill (Mossad *et al.*, 2016). Previous studies using electroencephalography have shown involvement of alpha and beta oscillatory power as well as parietal–occipital regional coupling in beta band in ToM conditions compared to control conditions (Guan *et al.*, 2018). In a study of joint attention, a precursor skill to ToM, comparing typically developing children and children with autism spectrum disorder, authors found that FT children showed increased beta oscillatory power in the temporal parietal junction prior to displaying joint attention while autism spectrum disorder children did not. In both groups, beta power was also related to mentalization scores, concordant with the notion of top-down processing. Similarly, in an electroencephalography study with a social attribution task, akin to the paradigm in this study, authors found theta, alpha and beta power were modulated by social interaction complexity. Therefore, our findings of reduced connectivity in the VPT group from 5 to 15 s in theta, alpha and beta (4–30 Hz) further corroborates the involvement of multiple cognitive processes underlying the social-cognitive abilities.

Findings of atypical MEG functional connectivity but typical fMRI source localization were surprising as clinical populations who have social-cognitive difficulties often show differences in functional specialization (Castelli, 2002). This discrepancy underscores the importance of using several methodological approaches when interrogating complex neuropsychological processes. In beta band, VPT children showed reduced connectivity in a network anchored in the right-angular gyrus (part of the temporal–parietal junction), the left superior temporal gyrus and included the medial pre-frontal cortex, the left inferior frontal gyrus, left amygdala and precuneus. The temporal–parietal junction, superior temporal gyri and medial pre-frontal cortex are reliably recruited in ToM processing (Carrington and Bailey, 2009) and are suggested to support distinct processes that underlie ToM. Reduced connectivity among these nodes in VPT children provides a novel hypothesis for the observed difficulties in social functioning. Increased connectivity in the VPT group during the social videos was found in the same frequency band (beta) in a small network anchored in the bilateral occipital lobes from 0 to 5 s. Occipital–cingulate connections in the first few seconds after stimulus onset suggest that the VPT children were pre-occupied with processing the visual information of the stimuli compared to FT children who were preferentially recruiting networks responsible for processing social stimuli. Thus, while they perceive a social situation to be more visually engaging, reduced connectivity in the right temporal parietal junction and medial pre-frontal cortex may make it more difficult to decide to which social interactions they should orient (Mitchell, 2008) and to accurately decipher the mental states of others.

During the same time window (5–10 s), VPT children showed reduced connectivity in a theta network that included hubs in the right superior temporal gyrus, the left orbital frontal gyrus, the right hippocampus and the right temporal pole. Von Stein and Sarnthein (2000) suggested that theta coherence modulates long-range communication with the frontal cortex sub-serving working memory processes. Doesburg, Vidal and Taylor (2013) found reduced theta band connectivity in children with autism spectrum disorder in a task-measuring cognitive flexibility. Here, we found that VPT children have reduced connectivity between the superior temporal and the orbital frontal gyri, suggesting that theta also regulates long-range neural communication in the social brain of FT children. Reduced connectivity in VPT children in alpha band was found in a network anchored in posterior regions, and has also been reported in VPT children in visual working memory tasks (Doesburg, Ribary, *et al.*, 2011; Sato *et al.*, 2018). Alpha synchrony supports attention and executive functioning (Palva and Palva, 2011), perceiving biological motion (Ulloa and Pineda, 2007) and social processes (Eidelman-Rothman *et al.*, 2016). Additionally, Levy *et al.* (2016) argued that the social brain processes information by modulating alpha oscillations to make certain social information more salient. Reduced connectivity in parietal and cingulate regions in alpha band likely contributes to VPT children attending either to irrelevant interactions or to misinterpreting interactions, consistent with our findings that errors on the SAT task were associated with reduced connectivity in alpha. Future studies in VPT children could investigate alpha-band synchrony using attention tasks to further test this question.

Although this study is the first multimodal investigation of social-cognitive processes in VPT children, there are some methodological limitations. This sample of VPT children was recruited at birth (*n* = 105), and each subject was contacted every 2 years. At each time point, all subjects who agreed to be contacted at their last visit were approached for the study. By 4 years, only 55 subjects returned to complete the neuroimaging and neuropsychology components. Therefore, at 8 years, the retention rate was 53%. Based on this phenomena, we cannot exclude the possibility that selection bias may be a limitation in this study, which perhaps higher functioning VPT children continued to agree to participate every 2 years. A consequence of this is that the sample of VPT children that contributed to the neuroimaging findings may not be representative of the initial VPT group recruited at birth on cognitive abilities, although the neonatal variables did not differ between those retained and those lost to follow-up. Another limitation which is common to other paediatric neuroimaging studies is loss of data due to head motion (Pang, 2011; Rapaport *et al*., 2019); head motion contributed to loss of data from six VPT and five FT subjects in the fMRI and two VPT and three FT subjects in the MEG.

## Conclusion

Despite mean IQ in the average range, there were significantly more VPT cases with elevated difficulties in social-cognition as reported by their parents. VPT children also made more errors in the interpretation of the social videos, often making inaccurate attributions. Although the fMRI results did not identify group differences, they were important in establishing that the ToM network is similar in children born VPT to their term-born peers. MEG results, however, showed marked distinctions in functional connectivity between VPT and FT children, indicating that more temporally sensitive measures may be required for studying high-level social-cognitive functions in clinical populations. MEG results demonstrated that VPT children had reduced connectivity in the ToM network while viewing shapes whose movement they correctly categorized as social movement. We therefore suggest that problems in social functioning in VPT children arise from bidirectional relationships between decreased connectivity in the ToM network and inaccurate interpretation of the social context. Since VPT children recruit a network involved in visual perceptual processing, interventions geared towards basic recognition of certain behaviours (i.e. playing and chasing), emotions (i.e. happy and angry) and personality traits are likely insufficient at 8 years. Instead, more complex strategies related to interpreting and evaluating outcomes of social situations would be more beneficial for this high-risk population.

## Supplementary material

Supplementary material is available at *Brain Communications* online.

## Supplementary Material

fcaa237_Supplementary_Data

## Abbreviations

ACG =: anterior cingulate gyrus
AEC =: amplitude envelope correlations
AMY =: amygdala
ANG =: angular gyrus
FFG =: fusiform gyrus
FT =: full-term born
fMRI =: functional magnetic resonance imaging
GA =: gestational age
HIP =: hippocampus
IFG =: inferior frontal gyrus
IOG =: inferior occipital gyrus
IPL =: inferior parietal lobule
IQ =: intelligence quotient
ITG =: inferior temporal gyrus
MEG =: magnetoencephalography
MFG =: middle frontal gyrus
MOG =: middle occipital gyrus
MTG =: middle temporal gyrus
OFC =: orbital frontal cortex
PCG =: posterior cingulate gyrus
SFG =: superior frontal gyrus
SPL =: superior parietal lobule
SRS =: social responsiveness scale
STG =: superior temporal gyrus
TP =: temporal pole
ToM =: Theory of Mind
VPT =: very preterm born
