# Supplementary material for: The preterm social brain: altered functional networks for Theory of Mind in very preterm children
Source: Brain Commun. 2021 Jan 25;3(1):fcaa237. doi: 10.1093/braincomms/fcaa237 (PMC7882208; doi:10.1093/braincomms/fcaa237)
Supplement: fcaa237_Supplementary_Data [file fcaa237_supplementary_data.docx]

**Supplementary Material**

Supplementary Table 1. Comparison between initial and returning cohorts on neonatal characteristics

|  | **Initial Cohort (n=105)** | **Returning at 8 years (n=40)** | ***t*** | ***p*-value** |
| --- | --- | --- | --- | --- |
| GA (Mean ± std. deviation) | 203.1±9.9 | 200.5±13.9 | 1.104 | 0.27 |
| Germinal matrix hemorrhage (Mean ± std. deviation) | 1.12 | 0.19 | 0.7626 | 0.45 |
| APGAR_5  (Mean ± std. deviation) | 7.15±1.7 | 7.36±1.9 | -0.57 | 0.57 |
| Neonatal Therapeutic Intervention Scoring system (NTISS)  (Mean ± std. deviation) | 16.11±2.9 | 16.46±3.7 | -0.52 | 0.60 |
| Clinical risk index for babies (CRIB II) (Mean ± std. deviation) | 6.75±2.4 | 7.08±2.8 | -0.6 | 0.53 |
| Score of Neonatal Acute Physiology (SNAP-II) (Mean ± std. deviation) | 6.96±7.6 | 7.08±9.5 | -0.05 | 0.95 |

Supplementary Table 2. Descriptions of a sample video depicting a social event and participant examples of errors

|  | Description |
| --- | --- |
| Response Example | Triangle is unable to open the door, so they ask the circle and diamond’s help. They all successfully open it together and go inside the box |
| Correct | The triangle cannot open the box. He needs help from the circle and square… tells him to help. He opens the box, put… and they all go outside the box. |
| Vague Reference | They’re opening the box, and the shapes are going in the boxes. |
| Misattribution | The triangle opens the square thing. And then the triangle puts the circle and the diamond in the square. |
| Inconsistent Proposition | The circle and square are helping triangle and they are bullying him |

Supplementary Table 3. Within group coordinates in the Social > Physical conditions
